# Global survey of physician testing practices for nontuberculous mycobacteria

**DOI:** 10.1183/23120541.00737-2022

**Published:** 2023-05-02

**Authors:** Michael R. Loebinger, Roald van der Laan, Marko Obradovic, Jakko van Ingen

**Affiliations:** 1 Royal Brompton Hospital and National Heart and Lung Institute, Imperial College London, London, UK; 2 Insmed BV, Utrecht, The Netherlands; 3 Insmed Germany GmbH, Frankfurt am Main, Germany; 4 Department of Medical Microbiology, Radboud University Medical Center, Nijmegen, The Netherlands

## Abstract

**Background:**

Certain patients are at greater risk of developing nontuberculous mycobacterial pulmonary disease (NTM-PD), including those with lung conditions such as bronchiectasis. Testing for nontuberculous mycobacteria (NTM) in patients at risk is necessary to identify NTM-PD and start appropriate management. The aim of this survey was to evaluate current testing practices for NTM and identify testing triggers.

**Methods:**

Physicians (n=455) who see at least one patient with NTM-PD in a typical 12-month period and test for NTM as part of practice from Europe, USA, Canada, Australia, New Zealand and Japan participated in a 10-min anonymised survey on NTM testing practices.

**Results:**

Bronchiectasis, COPD and use of immunosuppressants were the factors most likely to prompt testing among physicians in this survey (90%, 64% and 64%, respectively), with radiological findings the most common reason leading to considering NTM testing in patients with bronchiectasis and COPD (62% and 74%, respectively). Macrolide monotherapy in patients with bronchiectasis and inhaled corticosteroid use in patients with COPD were not important triggers for testing (15% and 9% of physicians, respectively). Persistent cough and weight loss triggered testing in >75% of physicians. Testing triggers were markedly different for physicians in Japan, with cystic fibrosis prompting testing in fewer physicians compared with other regions.

**Conclusions:**

Testing for NTM is influenced by underlying disease, clinical symptoms or radiological changes, but clinical practice varies considerably. Adherence to guideline recommendations for NTM testing is limited in certain patient subgroups and varies across regions. Clear recommendations on NTM testing are needed.

## Abstract 2

**Testing for nontuberculous mycobacteria is influenced by underlying disease, symptoms and radiological changes. For some patients, such as those initiating macrolide therapy, guidelines are not being followed and clear testing recommendations are needed.** https://bit.ly/3Km1l1X

## Introduction

Nontuberculous mycobacterial pulmonary disease (NTM-PD) is a rare, difficult-to-treat lung condition that is associated with a substantial patient burden [1–4]. The prevalence of NTM-PD is increasing globally [5] and recent predictions estimate many patients are undiagnosed [6, 7]. NTM-PD is associated with significant mortality [8–11], therefore timely diagnosis is important. However, NTM-PD can be challenging to diagnose because of a low index of suspicion and symptoms that are nonspecific and overlap with those of coexisting lung conditions [12, 13].

Certain patient groups have been demonstrated to have an increased risk of NTM-PD, such as those with underlying lung conditions, including cystic fibrosis (CF), non-CF bronchiectasis and chronic COPD, as well as those treated with immunosuppressants and systemic or inhaled steroids [14, 15]. Other commonly associated factors include low body mass index and thoracic skeletal abnormalities [16, 17]. Testing for nontuberculous mycobacteria (NTM) in patients at risk is important to identify NTM-PD early and start treatment in appropriate patients; early recognition, diagnosis and treatment may prevent disease progression [4, 18, 19].

A patient survey highlighted the need to raise awareness of NTM among all healthcare professionals, including primary care physicians, as well as the need for faster diagnosis and access to NTM-PD specialists [20]. A physician survey of perceived risk of NTM-PD in patients with bronchiectasis across Europe found physicians understand the risk of NTM-PD and associated morbidity in patients with bronchiectasis; however, most do not test for NTM-PD even in circumstances where guidelines recommend it [21]. The aim of the current survey was to evaluate current testing practices for NTM across geographies and identify testing triggers and adherence to current guideline recommendations.

## Methods

Physicians from Europe (UK, France, Germany, Italy, Spain and the Netherlands), USA, Canada, Australia, New Zealand and Japan were invited to participate in a 10-min anonymised survey on NTM testing practices *via* a secure online platform. Physicians invited to take part were recruited as part of the Sermo clinician networking platform (www.sermo.com) panel members and recruitment took place *via* e-mail. All panellists active in the past 365 days who were registered as practicing in the target specialties of respiratory medicine, pulmonary medicine, internal medicine or infectious diseases were invited to be screened on study eligibility. Inclusion criteria were set for the parameters of NTM caseload and testing; physicians were required to see at least one patient with NTM-PD in a typical 12-month period and requested or referred for NTM testing as part of their practice. Target sample sizes for each country were designed in consideration of 1) the estimated number of physicians seeing NTM within the target specialties and 2) specialty-specific response rates observed on prior surveys in the field of NTM. A breakdown of response rates observed in this study is presented in supplementary table S3.

The survey was conducted in the physician's native language. Physicians received an honorarium for completing the survey in line with current accepted standards. All registered panellist credentials were verified against bodies that maintain official registers of pulmonologists in each country. Sermo processes are in line with ISO 20252.

The survey instrument was developed in consultation with a range of stakeholders, including external experts (M.L. and J.v.I.) and Insmed BV (Utrecht, The Netherlands), and implemented by Porterhouse Insights (Reading, UK). Respondents were recruited by Porterhouse Insights. Informed consent was collected, in adherence with the British Healthcare Business Intelligence Association and Market Research Society and European Pharmaceutical Market Research Association guidelines [22, 23].

The survey was conducted between 6 August and 2 September 2021. The full survey questionnaire included five screening questions and 14 survey questions on NTM testing practice (supplementary tables S1 and S2). The survey data were analysed using contingency tables that were created using QPSMR version CL 64 2021.2c (www.qpsmr.org.uk). Descriptive statistics were employed looking at the subgroups and populations with the use of t-tests to highlight statistically significant differences.

## Results

### Survey respondent demographics

Overall, 455 physicians completed the survey across Europe (61% (n=276)), North America (23% (n=104)), Australasia (5% (n=25)) and Japan (11% (n=50)). The majority were in the specialty of pulmonology or respiratory medicine (63% (n=288)), with smaller numbers in internal medicine (11% (n=49)) and infectious diseases (25% (n=113)). A full breakdown of respondents’ countries and specialties is presented in table 1. Physicians’ caseloads varied, with 42% seeing >10 patients, 28% seeing 6–10 patients and 29% seeing ≤5 patients with NTM-PD in a typical 12-month period.

**TABLE 1:** Survey respondent demographics

**Country**	**Specialty**				**Total**
	**Pulmonology/respiratory medicine**	**Internal medicine**	**Infectious diseases**	**Other**	
**France**	33	3	14		50
**Germany**	28	10	11	1	50
**Italy**	26	10	14		50
**Spain**	33	4	13	1	51
**UK**	33	4	12	1	50
**The Netherlands**	20		5		25
**USA**	42		32	1	75
**Canada**	22	2	5		29
**Australia**	23		1		24
**New Zealand**	1				1
**Japan**	27	16	6	1	50
**Total**	288	49	113	5	455

Data are presented as n.

### Patient profiles prompting NTM testing

**FIGURE 1:**
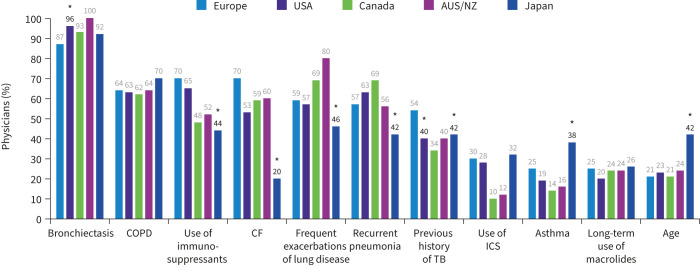
Factors that prompt testing for nontuberculous mycobacteria infection. AUS/NZ: Australia/New Zealand; CF: cystic fibrosis; TB: tuberculosis; ICS: inhaled corticosteroids. *: p<0.05 *versus* at least one other region.

**FIGURE 2:**
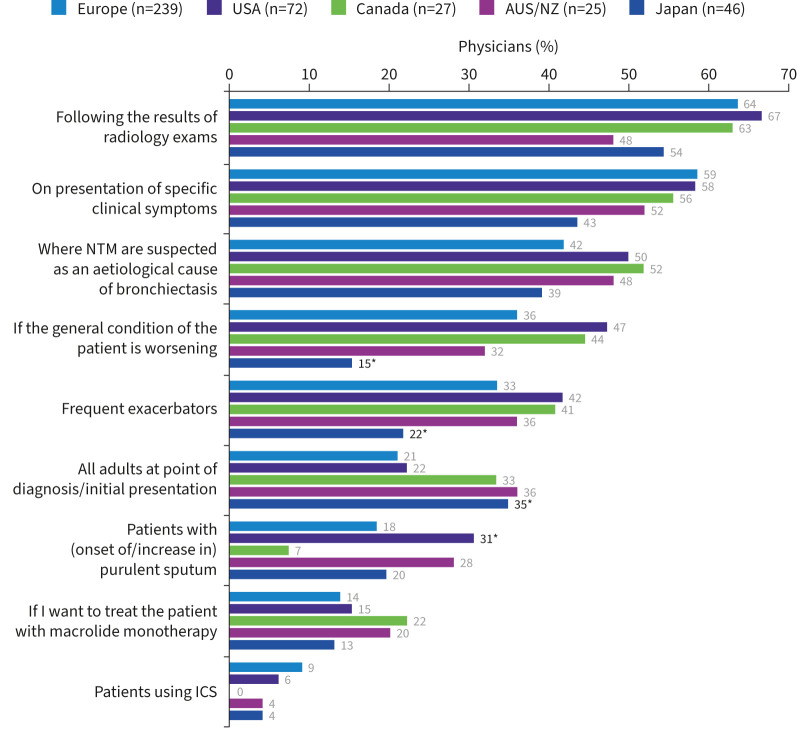
Reasons prompting testing for nontuberculous mycobacteria (NTM) infection in patients with bronchiectasis. AUS/NZ: Australia/New Zealand; ICS: inhaled corticosteroids. *: p<0.05 *versus* at least one other region.

Most regions were generally aligned regarding the patient profiles that prompted testing. Patients with bronchiectasis were the most frequently tested for NTM, with 90% of physicians globally considering testing patients with this condition (figure 1). For those who tested patients with bronchiectasis (n=409), testing was primarily considered following the results of radiological examination (62% (n=254)) or on presentation of specific clinical symptoms (such as weight loss or haemoptysis) (56% (n=230)), while few considered testing patients at initial presentation (24% (n=100)) (figure 2).

In contrast, macrolide use was rarely mentioned, with 24% of physicians overall considering testing for NTM in patients receiving long-term macrolide therapy (figure 1) and a similarly low number considered testing patients with bronchiectasis before initiating macrolide monotherapy (15% (n=61)) (figure 2). The number of physicians testing for NTM in patients with bronchiectasis using inhaled corticosteroids (ICS) was even lower (7% (n=28)).

**FIGURE 3:**
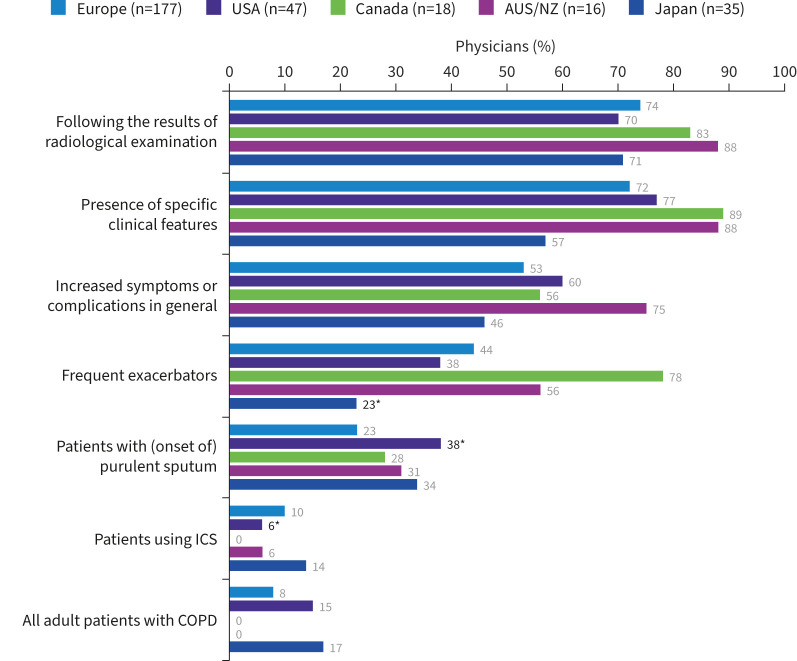
Reasons prompting testing for nontuberculous mycobacteria infection in patients with COPD. AUS/NZ: Australia/New Zealand; ICS: inhaled corticosteroids. *: p<0.05 *versus* at least one other region.

COPD and use of immunosuppressants were considered important, prompting testing in 64% of physicians globally (figure 1). In physicians who specified testing for NTM in patients with COPD (n=293), almost three-quarters considered testing following the results of radiological examinations (74% (n=218)) or on presentation of specific clinical symptoms (73% (n=213)) (figure 3). Few physicians considered testing all adults with COPD (9% (n=27)). There was greater variability observed between countries for frequent exacerbations as a trigger for NTM testing in patients with COPD, with 23% and 78% of physicians in Japan and Canada, respectively, reporting this as a relevant trigger.

**FIGURE 4:**
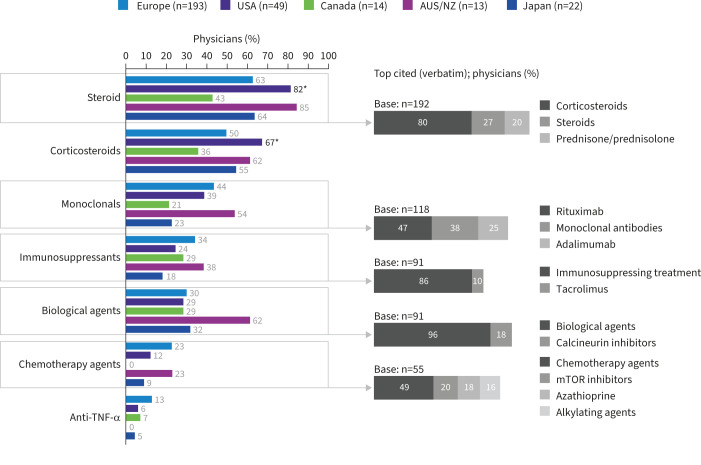
Immunosuppressant therapies prompting testing for nontuberculous mycobacteria infection. AUS/NZ: Australia/New Zealand; mTOR: mechanistic target of rapamycin; TNF: tumour necrosis factor. *: p<0.05 *versus* at least one other region.

Of physicians who specified testing for NTM in patients on immunosuppressants (n=291), 66% (n=192) of them consider testing for NTM in certain patients using steroids including corticosteroids (figure 4), yet few tested patients with COPD who were receiving ICS (9% (n=27)) (figure 3). Few physicians considered receiving anticancer agents (19%) or anti-tumour necrosis factor (TNF)-α inhibitors (10%) as important prompts to test for NTM.

Physicians in Japan considered different patient profiles for NTM testing compared with other regions. More physicians in Japan considered testing for NTM in patients with asthma (38% *versus* 24% globally) or age (42% *versus* 24% globally) compared with other cohorts (figure 1). Additionally, only 20% of physicians in Japan considered testing in patients with CF.

In contrast, CF was an important prompt for testing in all other regions (53–70% of physicians), particularly in European countries (70%). In the physicians who specified testing for patients with CF (60% (n=274)), half (50% (n=137)) considered testing all adults with CF. Physicians in Canada and Australia/New Zealand were more likely to consider testing all adults with CF compared with other regions (71% and 73%, respectively). Physicians in the USA and Japan were more likely to consider testing patients following the results of radiological examinations (50% and 60%, respectively) than testing all adults with CF (43% and 40%, respectively).

### Symptoms prompting NTM testing

**FIGURE 5:**
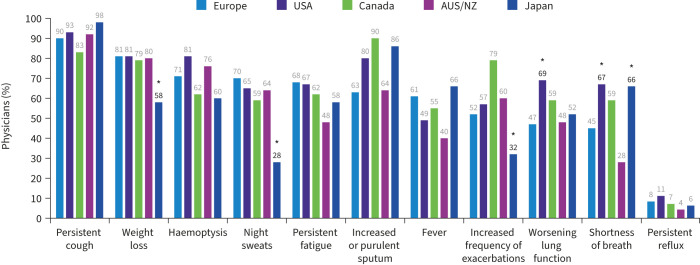
Clinical symptoms that prompt testing for nontuberculous mycobacteria infection. AUS/NZ: Australia/New Zealand. *: p<0.05 *versus* at least one other region.

Signs and symptoms that most commonly prompted NTM testing were persistent cough and weight loss, with >75% of physicians globally considering each of these symptoms as a trigger to test (figure 5). Assessing the correlations between primary symptoms and specific patient factors, such as underlying respiratory conditions, was not possible. Haemoptysis, increased or purulent sputum and fatigue were also frequently mentioned (71%, 70% and 66%, respectively), whereas physicians rarely considered testing for NTM important for patients with persistent reflux.

There was marked variability in the symptom profiles frequently prompting testing for NTM across regions, with physicians in Japan reporting different symptom profiles compared with other countries (figure 5). Physicians with larger NTM-PD caseloads (>10 patients annually) were significantly more likely (p<0.05) to test a patient with increased or purulent sputum, fever, increased exacerbation frequency or worsening lung function compared with those with smaller caseloads (≤10 patients annually).

### Risk factor combinations prompting NTM testing

**FIGURE 6:**
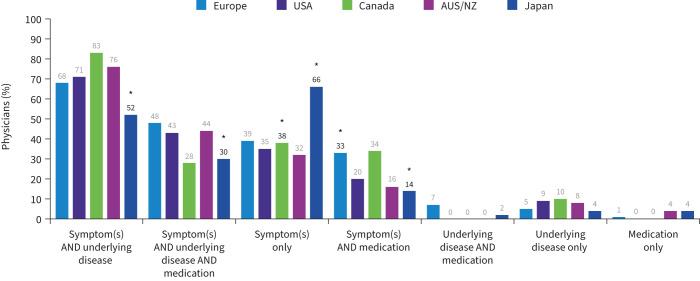
Combinations that prompt testing for nontuberculous mycobacteria infection. AUS/NZ: Australia/New Zealand. *: p<0.05 *versus* at least one other region.

The majority of physicians considered testing for NTM in patients presenting with a combination of symptoms and underlying disease, but generally underlying disease alone, with the exception of bronchiectasis, or medication alone rarely triggered NTM testing without the presence of clinical symptoms (figure 6). Combinations of symptoms without underlying disease or medication use were more likely to prompt testing for physicians in Japan compared with other regions. Respondents indicated they would test patients for NTM when a combination of symptoms or clinical conditions were present; on average five different risk factors were needed to prompt testing for NTM. Symptom profiles that included persistent cough with weight loss were the most frequently mentioned, with 62% of physicians mentioning this combination of symptoms in at least one of their patient profiles that would prompt testing for NTM. Physicians also frequently mentioned profiles including persistent cough with persistent fatigue (49% of physicians) as well as persistent cough with bronchiectasis (48%), followed by combinations containing weight loss with fatigue (47%) and persistent cough with sputum production (46%).

### NTM testing patterns

Upon worsening of clinical symptoms, most physicians repeated testing every 6 months. Physicians in Japan were more likely to not repeat microbiological testing compared with other regions (14% *versus* 3–5% of physicians, respectively) and perform radiological testing every 6 months (80% *versus* 54–67%, respectively). Physicians who saw >10 patients with NTM-PD per year were significantly more likely (p<0.05) to conduct microbiological testing every 6 months compared with those with ≤10 patients per year, but no differences were seen for radiological imaging between physicians managing different caseloads of NTM-PD patients.

Overall, mycobacterial sputum cultures were the tests most used to rule out NTM infection (91%). However, in Japan, high-resolution computed tomography scans and direct molecular tests were the more commonly used (76% and 74%, respectively), with mycobacterial sputum culture considered by 62% of physicians.

**FIGURE 7:**
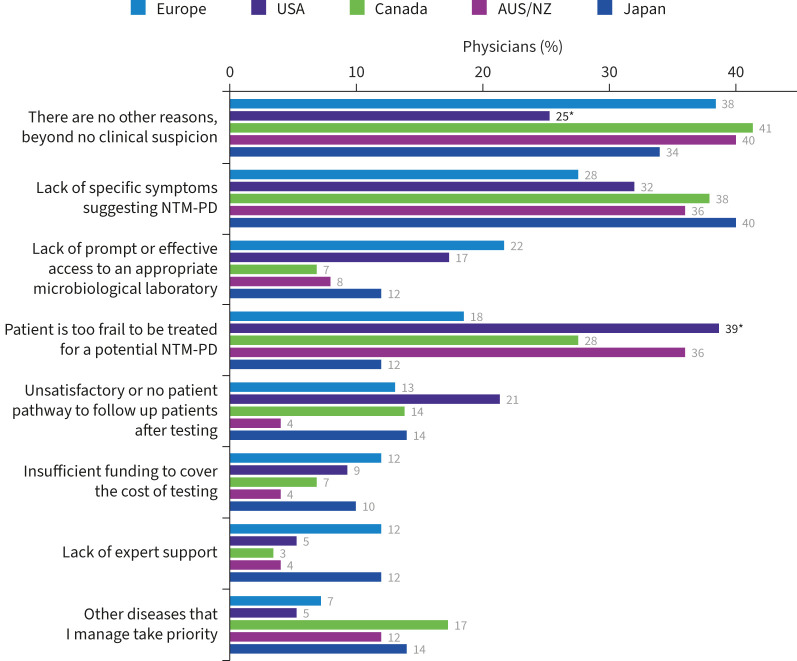
Reasons physicians choose not to test for nontuberculous mycobacteria infection, beyond that of no clinical suspicion. AUS/NZ: Australia/New Zealand; NTM-PD: nontuberculous mycobacterial pulmonary disease. *: p<0.05 *versus* at least one other region.

Reasons not to test for NTM were varied, with many physicians choosing not to test patients without specific symptoms suggesting NTM-PD. In the USA and Australia/New Zealand many physicians decide against testing for NTM because they believe their patient is too frail to receive treatment for NTM-PD (39% and 36%, respectively). Other reasons physicians cited for not testing for NTM included ones related to lack of sufficient infrastructure, *e.g.* lack of access to microbiological laboratories (22%), insufficient funding (12%) and lack of expert support (12%) in Europe, whereas 21% of physicians in the USA did not test because there was no satisfactory patient pathway to refer or manage patients after testing (figure 7).

## Discussion

Results from this survey indicate a high threshold for NTM testing among physicians globally, but it is clear that certain symptoms, radiological changes and predisposing conditions are important drivers to test for NTM. Persistent cough and weight loss were key clinical symptoms prompting testing for respondents, suggesting that they are commonly testing patients with more advanced disease. The patients considered for testing most often were those with bronchiectasis followed by COPD, with radiological features specifically being the most common reason for NTM testing in these patients. However, despite growing evidence [14, 24, 25] and/or guideline recommendations, macrolide monotherapy in patients with bronchiectasis and ICS use in patients with COPD were not important triggers for testing in this survey.

There were marked differences in the symptom and disease profiles that prompted testing among physicians in Japan compared with other regions, particularly for CF, which was considered an important risk factor to prompt testing by only 20% of physicians in Japan compared with 60% globally. However, CF is rare in Asian populations and physicians in Japan are less likely to see these patients, potentially leading to CF being considered less relevant for testing for these physicians.

Guidelines on NTM testing for patients with predisposing conditions are limited to bronchiectasis and CF. Regional bronchiectasis guidelines, including the European Respiratory Society guidelines, recommend testing for NTM when NTM is suspected as the cause of bronchiectasis or at initial evaluation, in patients with radiological or clinical features of NTM-PD, or prior to initiating long-term antibiotic therapy such as macrolide monotherapy [26–32]; some guidelines also recommend regularly testing all patients with bronchiectasis, where possible [26, 29–31]. CF recommendations specify that cultures for NTM should be performed annually in spontaneously expectorating individuals with a stable course [33]. No guidelines or recommendations specifically address testing for NTM in patients with COPD.

Findings from this survey showed that 90% of physicians considered testing patients with bronchiectasis for NTM, most commonly following the results of a radiology examination or on presentation of specific clinical symptoms, in line with guideline recommendations. These findings support recent reports by Wagner *et al*. [21], who demonstrated in a European survey that 85% of physicians tested at least some of their patients with bronchiectasis. Data from the EMBARC registry also highlighted wide regional variation in testing frequency in the UK, ranging from 8.3% to 35.5% of patients with bronchiectasis [34]. In the current survey, 60% of physicians considered testing patients with CF and only 50% of these tested all adults with CF despite guideline recommendations. Patients with CF are often treated in specialised CF centres and therefore physicians who participated in the survey may not see these patients or only see them as part of shared care constructions, leading them to be less likely to consider these patients for NTM testing. It was notable that in this patient group in the USA and Japan testing was more likely to be considered after suspicions raised following radiology examinations. These results may suggest that radiological examinations in vulnerable patients are undertaken regularly in these countries.

Guideline recommendations are frequently not being followed with respect to which patients should be tested: in this survey 24% of physicians globally tested patients for NTM at initial presentation of bronchiectasis and very few physicians tested patients with bronchiectasis prior to receiving long-term macrolide monotherapy (15%). Results are similar to a survey of testing practice in patients with bronchiectasis in the Asia-Pacific region that noted that 21% of physicians performed acid-fast bacilli smear tests and cultures at the initial visit [35]. Findings from previous surveys also report low levels of testing in patients with bronchiectasis prior to initiating long-term macrolide therapy [21, 34]. Macrolides are the backbone of NTM-PD therapy and macrolide monotherapy in the presence of NTM infection is a risk factor for macrolide resistance [25, 36]; therefore, testing patients for NTM when considering use of macrolides should be undertaken, in line with guidelines, to retain possible future treatment options.

The decision to test for NTM should consider the overall patient profile and is usually based on a combination of factors, rather than individual clinical symptoms or patient types. Patients receiving immunosuppressants who also present with respiratory symptoms or have predisposing conditions may be at risk of NTM-PD and testing is warranted. For example, it is well established that anti-TNF-α therapies are associated with an increased risk for NTM disease including NTM-PD and experts advise a low threshold to test for NTM in these patients [37, 38]. In this survey, physicians were most likely to consider testing for NTM in patients presenting with combinations of symptoms that included persistent cough and weight loss, suggesting many physicians look for at least one of these symptoms before testing or they are the most consistently presented symptoms in patients. Physicians generally did not consider testing for NTM in patients with underlying disease or using certain medications without the presence of clinical symptoms suggestive of NTM-PD and physicians considered clinical symptoms in combination with underlying disease to be the most important prompt for testing. It is likely that certain symptoms prompt testing primarily in patients with other indicators of clinical suspicion for NTM-PD, *e.g.* underlying bronchiectasis. Survey data cannot explore in detail correlations between multiple symptoms prompting clinical suspicion and in which patients.

Experts who see many patients with NTM-PD are more likely to have their clinical suspicion of NTM infection raised following a broad range of, often, nonspecific or diffuse symptoms, with the most common suspicious symptoms including persistent cough, haemoptysis, night sweats, weight loss, persistent fatigue, increased or purulent sputum, fever and an increase in exacerbation frequency of their underlying condition. Use of ICS in patients with COPD, anti-TNF-α therapies and persistent reflux have been linked to an increased risk for NTM-PD and are also considered important prompts to test by experts [14, 15, 39]. These factors were considered less important by physicians in the survey, suggesting clear disparities between physician practice and expert opinion.

These survey data highlight different approaches of physicians to NTM testing, influenced by factors such as access to mycobacteriological laboratories or in some cases because respondents believed testing was not useful in patients they deemed too frail to complete the challenging and lengthy treatment regimens required to treat NTM-PD appropriately. Tolerable, shorter and more effective treatments are urgently needed to lower the threshold to treat. In addition, supportive therapies aimed at airway clearance may provide benefit for patients deemed too frail for antibiotic therapies, providing an argument to test and diagnose even in those too frail to treat [40]. Importantly, some of the testing approaches identified in this survey suggest that some patient groups may be missing out on appropriate testing.

Limitations inherent to surveys and their design, with a risk of selection and perception bias in the questionnaire, apply to this research. The combination of symptoms and risk factors that lead physicians to test a particular patient for NTM can be considered as subjective and can be difficult to capture in surveys in general. In addition, survey participation was limited to physicians who see at least one patient with NTM per year and who test for NTM as part of clinical practice. It would be important to survey NTM testing practices in physicians who do not manage patients with NTM-PD, as it is likely that this group does not test appropriately, and may even further highlight the disparities in testing practices between physicians and experts who frequently see patients with NTM-PD.

In conclusion, testing for NTM is influenced by underlying disease and the presence of clinical symptoms or radiological changes. However, clinical practice varies considerably across geographies and is not aligned with existing recommendations for NTM testing in certain patient subgroups. The symptoms that often trigger testing for NTM are those seen in later-stage NTM-PD, which can potentially lead to late diagnoses of NTM-PD with a negative impact on patient outcomes. Including NTM-PD in other respiratory disease guidelines, such as COPD, with clear recommendations on NTM testing, monitoring and management in these patient groups is warranted.

## Supplementary material

10.1183/23120541.00737-2022.Supp1 **Please note:** supplementary material is not edited by the Editorial Office, and is uploaded as it has been supplied by the author.
Supplementary material 00737-2022.SUPPLEMENT
