# Supplementary material for: Global survey of physician testing practices for nontuberculous mycobacteria
Source: ERJ Open Res. 2023 May 2;9(3):00737-2022. doi: 10.1183/23120541.00737-2022 (PMC10152245; doi:10.1183/23120541.00737-2022)
Supplement: Supplementary file 1 [file 00737-2022.SUPPLEMENT.pdf]

Supplementary materials

Supplementary Table S1. Screening questions

	SCREENING		
#	Question	Code	Route
S0	Country		BASE =ALL AUTO CODE
		France	1
		Germany	2
		Italy	3
		Spain	4
		UK	5
		Netherlands	6
		USA	7
		Canada	8
		Australia	9
		New Zealand	10
		Japan	11
S1	Do you <u>see</u> patients with **non-tuberculous mycobacterial (NTM) lung disease** as part of your practice?		SINGLE CODE
	Yes	1	→ CONTINUE
	No	2	→ CLOSE
S2	How many patients with non-tuberculous mycobacterial (NTM) lung disease do you **typically <u>see</u> in a 12 month period**?		SINGLE CODE
	Zero patients/none	1	→ CLOSE
	1 patient	2	→ CONTINUE
	2–5 patients	3	→ CONTINUE
	6 to 10 patients	4	→ CONTINUE
	More than 10 patients	5	→ CONTINUE
S3	And do you <u>test</u> patients for non-tuberculous mycobacterial (NTM) lung disease as part of your practice?		SINGLE CODE
	Yes	1	→ CONTINUE
	No	2	→ CLOSE
S4	What is your primary medical specialty?<br>Select one that best applies		SINGLE CODE
	Pulmonology/Respiratory medicine	1	→ CONTINUE
	Internal Medicine	2	→ CONTINUE
	Infectious Diseases	3	→ CONTINUE
	Other *please write in*____________________	4	→ CONTINUE
S5	**ASK IF INTERNAL MEDICINE ONLY:**<br>Do you have a secondary specialty in pulmonology?		
	Yes		
	No	1	→ CONTINUE
		2	→ CONTINUE

	RESPONDENT MUST SEE AND TEST NTM-LUNG DISEASE PATIENTS

Supplementary Table S2. Survey questionnaire

	SECTION A		
#	Question	Code	Route
A1a	Which clinical symptoms would prompt you to test for NTM infection in a patient?<br><br>*Check those that apply and/or write in any others in the box below*<br>Persistent cough<br>Weight loss<br>Increased or purulent sputum<br>Fever<br>Persistent fatigue<br>Shortness of breath<br>Night sweats<br>Worsening lung function<br>Haemoptysis<br>Increased frequency of exacerbations<br>Gastroesophageal reflux<br>Other clinical symptoms<br>*please specify – write in*__________________<br>*please use 1 row per symptom*____________<br>____	**1**<br>**2**<br>**3**<br>**4**<br>**5**<br>**6**<br>**7**<br>**8**<br>**9**<br>**10**<br>**11**<br>**12**<br>**13**<br>**14**	**MULTI CODE**
	**IF A1 = 8 ASK A1B**		
A1b	You indicate that “*worsening lung function*” is a clinical symptom that would prompt you to test for NTM infection in a patient – at what level of decline would you begin to test on this basis?<br><br>**FEV1 & FVC**<br>% decline of:<br>Less than 10%<br>10–20%<br>21–30%<br>More than 30%<br>Other please write in_____________________	1<br>2<br>3<br>4<br>5	**SINGLE CODE**

A1c	In patients that you test for NTM infection based on worsening clinical symptoms how often would you repeat sputum (microbiological) testing or radiological imaging?<br><br>Do not repeat test<br>Every 6 months<br>Annually<br>Every 2 years<br>Every 5 years<br>Other<br>please specify:___________________________	**Microbiological testing**<br><br><br><br><br>1<br>2<br>3<br>4<br>5<br>6	**Radiological imaging**<br><br><br><br><br>1<br>2<br>3<br>4<br>5<br>6
	**ASK ALL**		
A2a	Which of the following specific patient types, if any, do you test or monitor for NTM infection?<br><br>*Check all that apply*<br>COPD<br>Bronchiectasis<br>Asthma<br>Cystic fibrosis (CF)<br>Age<br>Use of immunosuppressant therapies (e.g. biologicals or immunosuppressant drugs)<br>Use of inhaled corticosteroids<br>Use of long-term macrolide antibiotics<br>Patients with a previous history of tuberculosis (TB)<br>Patients with recurrent pneumonia<br>Patients with recurrent and frequent exacerbations of their underlying lung condition<br>None **(EXCLUSIVE) – SKIP TO A16**	 <br><br><br>**1**<br>**2**<br>**3**<br>**4**<br>**5**<br>**6**<br><br>**7**<br>**8**<br>**9**<br><br>**10**<br>**11**<br><br><br>**12**	**MULTI CODE**
A2b	**IF MORE THAN 5 CODES SELECTED AT PREVIOUS QUESTION, ASK:**<br>And of these, which 5 do you consider to be the most important to test or monitor for NTM infection?<br><br>**INSERT CODES SELECTED FROM PREVIOUS QUESTION, AND MULTICODE 5 OF THEM**<br>➔ **THESE CODES TO BE CARRIED THROUGH TO QUESTION LOOPS TO FOLLOW**		
**PATIENT TYPE LOOPED QUESTIONS**			
	**IF A2a = 1 ASK A3 QUESTIONS ON ONE SCREEN IF POSSIBLE**		

A3a	Which COPD patients do you test or monitor for NTM infection?<br><br>*Check all that apply*<br>All adult patients with COPD<br>Following the results of radiological examination – physical features in the lung that lead to suspicion of NTM infection<br>Presence of specific clinical features (such as weight loss, haemoptysis) that lead to suspicion of NTM infection<br>Increased symptoms or complications in general that lead to suspicion of NTM infection<br>Frequent exacerbators<br>Patients with (onset of) purulent sputum<br>Patients in receipt of ICS<br>Other COPD patient type<br>*Please write in*___________________________	 <br><br>**1**<br>**2**<br><br><br>**3**<br><br><br>**4**<br><br><br>**5**<br><br>**6**<br>**7**<br>**8**	**MULTI CODE- EXCEPT CODE '1' – EXCLUSIVE CODE**
A3b	And is severity in COPD a rationale for testing or monitoring for NTM infection?<br>No<br>Yes<br>– at GOLD stage I<br>– at GOLD stage II<br>– at GOLD stage III<br>– at GOLD stage IV	 <br>1<br><br>2<br>3<br>4<br>5	**SINGLE CODE**<br><br>**IF YES CODED – POP UP SECOND PART OF ANSWER AND REQUIRE CODE**
	**IF A2a = 2 ASK A4 QUESTIONS ON ONE SCREEN IF POSSIBLE**		

A4a	Which bronchiectasis patients do you test or monitor for NTM infection?<br><br>*Check any of the following statements that apply*<br><br>All adult patients with bronchiectasis are tested at point of diagnosis/initial presentation<br>Following the results of radiology exams, showing physical features of the lung which lead to the suspicion of NTM infection<br>On presentation of specific clinical symptoms (such as weight loss, haemoptysis) which lead to the suspicion of NTM infection<br>Where NTM are suspected as an aetiological cause of bronchiectasis<br>If I want to treat the patient with macrolide monotherapy<br>Frequent exacerbators<br>If the general condition of the patient is worsening<br>Patients with (onset of/increase in) purulent sputum<br>Patients in receipt of ICS<br>Other bronchiectasis patient type<br>*Please write in*__________________________	**1**<br>2<br>3<br>4<br>5<br>6<br>7<br>8<br>9<br>10	**MULTI CODE- EXCEPT CODE '1' – EXCLUSIVE CODE**
A4b	And is severity in bronchiectasis a rationale for testing or monitoring for NTM infection?<br>No<br>Yes<br>- 0 - 4 points*<br>- 5 - 8 points*<br>- ≥ 9 Points*<br><br>*Severity criteria using the Bronchiectasis Severity Index[1]<br>1. EMBARC. Bronchiectasis Severity Index. https://www.bronchiectasis.eu/severity-assessment [Accessed June 2021]	**1**<br>**2**<br>**i**<br>**ii**<br>**iii**	**SINGLE CODE**<br><br>**IF YES CODED – POP UP SECOND PART OF ANSWER AND REQUIRE CODE**
	**IF A2a = 3 ASK A5 QUESTIONS ON ONE SCREEN IF POSSIBLE**		

A5a	Which asthma patients do you test or monitor for NTM infection?<br><br>*Check any of the following statements that apply*<br><br>All adult patients with asthma are tested at point of diagnosis/initial presentation<br>Following the results of radiology exams, showing physical features of the lung which lead to the suspicion of NTM infection<br>On presentation of specific clinical symptoms (such as weight loss, haemoptysis) which lead to the suspicion of NTM infection<br>If I want to treat the patient with macrolide monotherapy<br>Frequent exacerbators<br>If the general condition of the patient is worsening<br>Patients with (onset of/increase in) purulent sputum<br>Patients in receipt of ICS<br>Other asthma patient type<br>*Please write in*___________________________	 <br><br><br>1<br>2<br>3<br>4<br>5<br>6<br>7<br>8<br>9	**MULTI CODE- EXCEPT CODE '1' – EXCLUSIVE CODE**
A5b	And is severity in asthma a rationale for testing or monitoring for NTM infection?<br>No<br>Yes<br>– intermittent<br>– mild persistent<br>– moderate persistent<br>Severe persistent<br>Status asthmaticus	 <br>1<br>2<br>i<br>ii<br>iii<br>iv<br>v	**SINGLE CODE**<br><br><br><br><br>**IF YES CODED – POP UP SECOND PART OF ANSWER AND REQUIRE CODE**
	**IF A2a = 4 ASK A6 QUESTIONS ON ONE SCREEN IF POSSIBLE**		

A6a	Which CF patients do you test or monitor for NTM infection?<br><br>*Check any of the following statements that apply*<br><br>All adults with CF are tested<br>Following the results of radiology exams, showing physical features of the lung which lead to the suspicion of NTM infection<br>On presentation of specific clinical symptoms (such as weight loss, haemoptysis) which lead to the suspicion of NTM infection<br>If the general condition of the patient is worsening<br>Other CF patient type<br>*Please write in*________________________	1<br>2<br>3<br>4<br>5	**MULTI CODE- EXCEPT CODE ‘1’ – EXCLUSIVE CODE**
	**IF A2a = 6 ASK A8 QUESTIONS ON ONE SCREEN IF POSSIBLE**		
A8a	Which patients do you test or monitor for NTM infection based on age?<br><br>*Check the following categories that apply*<br><br>Those under 30 years old<br>Those between 30 and 50 years old<br>Those between 51 and 60 years old<br>Those between 61 and 70 years old<br>Those between 71 and 80 years old<br>Those over 80 years old	**1**<br>**2**<br>**3**<br>**4**<br>**5**<br>**6**	**MULTI CODE**
	**IF A2a = 7 ASK A9 QUESTIONS ON ONE SCREEN IF POSSIBLE**		
A9a	Which patients do you test or monitor for NTM infection based on their use of immunosuppressant therapies the include:<br>• IV/oral corticosteroids;<br>• biologics such as adalimumab, rituximab or others;<br>• monoclonal antibodies such as basiliximab, daclizumab, trastuzumab;<br>• immunosuppressant drugs including alkylating agents, folic acid antagonists, mTOR inhibitors, calcineurin inhibitors, Janus kinase inhibitors among others<br><br>Please specify the drugs that prompt you to test for NTM infection<br>Write in______________________<br>Write in______________________		**OPEN TEXT ALLOW UP TO FIVE ROWS**
	**IF A2a = 10 ASK A10 QUESTIONS ON ONE SCREEN IF POSSIBLE**		

A10a	Which patients with a previous history of tuberculosis (TB) do you test or monitor for NTM infection?<br><br>*Check any of the following statements that apply*		**MULTI CODE- EXCEPT CODE ‘1’ – EXCLUSIVE CODE**
	All adults with previous history of TB are tested	1	
	Following the results of radiology exams, showing physical features of the lung which lead to the suspicion of NTM infection	2	
	On presentation of specific clinical symptoms (such as weight loss, haemoptysis) which lead to the suspicion of NTM infection	3	
	If the general condition of the patient is worsening	4	
	**NOW ASK ALL**		
A11a	In this question we are looking to explore which combination of symptoms/risk factors you would see in a patient that would increase your suspicion they might have NTM-PD and lead you to test for infection.<br><br>*Please enter one combination per column*<br>*Combinations should be realistic, but may contain as many features you are likely to see together in clinical practice*<br>*Please use as many of the columns as you need, but at least 1*		

	**Combination**	Patient 1 **MUST PROVIDE SOME SELECTION**	Patient 2 **OPTIONAL**	Patient 3 **OPTIONAL**	Patient 4 **OPTIONAL**	Patient 5 **OPTIONAL**
	Persistent cough	○	○	○	○	○
	Weight loss	○	○	○	○	○
	Onset of/increase in purulent sputum	○	○	○	○	○
	Fever	○	○	○	○	○
	Persistent fatigue	○	○	○	○	○
	Shortness of breath	○	○	○	○	○
	Night sweats	○	○	○	○	○
	Worsening lung function	○	○	○	○	○
	Haemoptysis	○	○	○	○	○
	Increased frequency of exacerbations	○	○	○	○	○
	Gastroesophageal reflux disease and/or use of acid suppression medication (e.g. PPI)					
	Underlying disease – bronchiectasis					

	Underlying disease – COPD					
	Underlying disease – asthma					
	Underlying disease – CF					
	Underlying disease – history of TB					
	Morphological features (e.g. taller than average, elongated arm span >1.03 of individual's height, elderly [male or female] presence of pectus excavatum or scoliosis)					
	Medication use – oral corticosteroids					
	Medication use – IV corticosteroids					
	Medication use – Biologics (e.g. abatacept, rituximab, etc.)					
	Medication use – monoclonal antibodies (e.g. basiliximab, daclizumab)					
	Medication use – interferons (any type)					
	Medication use – Immunosuppressants (all classes e.g. mTOR inhibitors, calcineurin inhibitors, Janus kinase inhibitors etc.)					
	History of NTM isolation/disease					
	Other (please specify)					
A12	And are there any other reasons (other than those previously outlined) that would prompt you to test or monitor for NTM infection?<br>No<br>Yes<br>IF YES please specify reasons:_____________ ________			1<br>2	**SINGLE CODE – IF YES SHOW OPEN TEXT BOX AND REQUIRE AT LEAST 5 CHARACTERS**	

	ASK ALL		
A13	Which tests do you undertake to rule out NTM infection?<br><br>Direct molecular tests for NTM organisms on primary sample<br>Mycobacterial sputum culture<br>Other microbiological tests<br>please specify: ______________________<br>________<br>Lung function tests<br>High resolution CT scan<br>Other tests<br>please specify:______________________	 <br><br>1<br><br>2<br>3<br><br><br>4<br>5<br>6	MULTI CODE
A14	Are any of the below points reasons why you would not test for NTM infection, beyond that of no clinical suspicion?<br>*Please select as many that apply to you*<br><br>Insufficient funding to cover the cost of testing<br>Lack of prompt or effective access to an appropriate microbiological laboratory<br>Unsatisfactory or no patient pathway to follow up patients after testing<br>Lack of expert support<br>Other diseases that I manage take priority<br>Patient is too frail to be treated for a potential NTM-PD<br>Lack of specific symptoms suggesting NTM<br>Other reason<br>please specify ______________________<br>There are no other reasons (EXCLUSIVE)	 <br><br><br><br>1<br>2<br><br>3<br><br>4<br>5<br>6<br>7<br><br>8	MULTI CODE
	THANK AND CLOSE		
	AE REPORTING LINK SET UP FOR OPEN ENDED TEXT<br>WE WILL NEED ACCESS TO REVIEW FREE TEXT RESPONSES TO CHECK FOR ADVERSE EVENTS & REPORT WITHIN 1 BUSINESS DAY OF THE RESPONSE		

Supplementary Table S3. Survey response rates

Country	Number of physicians invited	Number of physicians completing the screener	Non-response rate (%)
France	557	52	91
Germany	697	63	91
Italy	251	56	78
Spain	322	53	84

UK	572	55	90
The Netherlands	288	28	90
USA	134	78	42
Canada	96	30	69
Australia	724	25	97
New Zealand	72	1	99
Japan	351	56	84
Total	4064	497	88
